# Identification of a New Badnavirus in the Chinaberry (*Melia azedarach*) Tree and Establishment of a LAMP-LFD Assay for Its Rapid and Visual Detection

**DOI:** 10.3390/v13122408

**Published:** 2021-12-01

**Authors:** Huixin Lu, Jintian Tang, Kai Sun, Xiaoping Yu

**Affiliations:** Zhejiang Provincial Key Laboratory of Biometrology and Inspection & Quarantine, College of Life Sciences, China Jiliang University, Xueyuan Street, Xiasha Higher Education District, Hangzhou 310018, China; lhx20811@163.com (H.L.); jintiantang@cjlu.edu.cn (J.T.)

**Keywords:** chinaberry tree, high throughput sequencing, badnavirus, LAMP-LFD

## Abstract

The Chinaberry tree, a member of the *Meliaceae* family, is cultivated in China for use in traditional medicines. In 2020, Chinaberry trees with leaf deformation symptoms were found in Hangzhou, Zhejiang province, China. In order to identify possible pathogenic viruses, a symptomatic sample was subjected to deep sequencing of small interfering RNAs. Assembly of the resulting sequences led to the identification of a novel badnavirus, provisionally designated Chinaberry tree badnavirus 1 (ChTBV1). With the recent development of China’s seedling industry and increasing online shopping platforms, the risk of tree virus transmission has increased substantially. Therefore, it is important to detect the occurrence of ChTBV1 to ensure the safety of the Chinaberry tree seedling industry. Here, we describe the development and validation of a sensitive and robust method relying on a loop-mediated isothermal amplification (LAMP) assay, targeting a 197 nt region, to detect ChTBV1 from Chinaberry tree leaves. The LAMP assay was also adapted for rapid visualization of results by a lateral flow dipstick chromatographic detection method.

## 1. Introduction

*Melia azedarach* Linn., commonly known as Chinaberry, Persian Lilac, and Umbrella Tree, is a deciduous tree in the family *Meliaceae* [1]. It is native to Australia and South East Asia and is distributed in the Sichuan, Yunnan, Guizhou, Jiangsu, and Zhejiang Provinces of China. As a Chinese traditional medicine, the whole plant or its specific parts (leaves, stems, and roots) are commonly used to treat diarrheal, diabetic, rheumatic, and hypertensive diseases [2]. Studies on *M. azedarach* have mainly focused on its cultivation and medicinal activity, but viruses infecting the Chinaberry tree have not yet been identified in China. In 2020, a survey of viral diseases was carried out on plants used in traditional Chinese medicines in Zhejiang province, and here we report the identification and characterization of a novel badnavirus provisionally designated Chinaberry tree badnavirus 1 (ChTBV1) from deformed leaves of Chinaberry tree.

Members of the genus *Badnavirus* in the family *Caulimoviridae* are plant pararetroviruses with a 7–9 kbp single circular double-stranded DNA genome and non-enveloped bacilliform particles of 120–150 ± 30 nm [3]. Badnaviruses cause plant diseases of varying severity in several economically important hosts, such as rice tungro, banana streak, and cacao swollen shoot [4]. In recent years, several new badnaviruses have been identified and found to be prevalent in a variety of plants. We also found that ChTBV1 infections affect seedling growth and provide possible problems for the Chinaberry seedling industry.

Because ChTBV1 may cause substantial forestry losses, developing a rapid and simple assay for ChTBV1 detection is urgent. Based on our knowledge of the molecular biology of ChTBV1, we combined the loop-mediated isothermal amplification (LAMP) technique with a lateral flow dipstick (LFD) detection tool to provide simultaneous visual detection of ChTBV1. The LAMP technique has been widely applied to detect plant viruses, including Tomato leaf curl New Delhi virus [5], Apple chlorotic leaf spot virus [6], and Sugarcane streak mosaic virus [7]. The technique is based on the Bst DNA polymerase large fragment with both high strand displacement and replication activities that permit amplification of traces of nucleic acid under isothermal conditions (60–65 °C) [8]. Recently, a chromatographic LFD format was applied to determine the LAMP results during the Citrus leaf blotch virus detection [9]. LFD provides a rapid and simple method that does not require special instruments to visualize amplification [10]. The method relies on labeled amplification products that are bound by antibodies on a test strip that produces a color reaction within 2 min of the sample application and eliminates equipment dependence and contact with DNA dyes.

In the present study, we report a novel badnavirus provisionally designated ChTBV1 using Next-Generation Sequencing. Furthermore, we developed a rapid and visual detection method for ChTBV1 based on LAMP-LFD. We believe that the genomic analysis and the newly established LAMP-LFD method could provide early warning of ChTBV1 infections to benefit tree production and ensure forestry security.

## 2. Materials and Methods

### 2.1. Plant Materials and High Throughput Sequencing (HTS)

Leaves showing deformation symptoms were collected from *M. azedarach* in Zhejiang, China. For small RNA (sRNA) sequencing, total RNA was purified using TRIzol according to the manufacturer’s instructions (Invitrogen, Carlsbad, CA, USA). The resulting RNA samples were frozen in liquid nitrogen, preserved in carbon dioxide ice blocks, and shipped to Majorbio Biomedical Technology Co., Ltd. (Shanghai, China) for sRNA deep sequencing with a NovaSeq 6000 sequencer (Illumina, Inc., San Diego, CA, USA).

Raw Illumina sRNA reads were “cleaned” with Trim Galore v0.3.7 [11] to remove adaptor sequences and discard reads smaller than 16 nucleotides (nt), longer than 30 nt, and reads containing low-quality tags. Virus contigs were assembled and identified from sRNA sequences using VirusDetect pipeline v1.6, which uses Velvet for de novo assembly of small RNAs to compare contigs in the reference database for virus identification [12].

### 2.2. Virus Identification

In order to determine the complete viral genome sequence, overlapping primer pairs (Supplementary Table S1) were designed based on ChTBV1 contigs. Total DNA was extracted from plants using a standard CTAB method [13]. The viral genome was amplified by polymerase chain reaction (PCR) using high-fidelity DNA polymerase KOD-Plus-Neo (Toyobo, Osaka, Japan) to produce 6 overlapping fragments encompassing the complete ChTBV1 genome. PCR products were purified and cloned into a pLB-Blunt cloning kit (Tiangen, Beijing, China). At least five clones for each amplicon were sequenced, and the complete ChTBV1 genome was assembled with the Seqman component of the Lasergene package (DNASTAR, Madison, WI, USA).

### 2.3. Genome and Phylogenetic Analysis

The BLAST program at the National Center for Biotechnology Information (NCBI) was used to analyze the complete viral sequence to identify sequences closest to ChTBV1. Open reading frames (ORFs) were identified using ORF Finder (http://www.ncbi.nlm.nih.gov/projects/gorf) (accessed on 1 June 2021), and multiple sequence alignments of the complete ChTBV1 genome were compared with gene-specific nucleotides of other badnaviruses using the MATTF alignment program (https://www.ebi.ac.uk/Tools/msa/mafft) (accessed on 1 June 2021). A neighbor-joining phylogenetic tree was constructed from the alignment of ChTBV1 genome sequences and other representative badnavirus sequences using MEGA v7.0 software [14] with 1000 bootstrap replicates.

### 2.4. Design of LAMP Primers

LAMP primers were designed with Primer Explorer V5 software (http://primerexplorer.jp/e/) (accessed on 1 July 2021) using a partial ORF3 sequence. LAMP primer information and positions are shown in Table 1 and Supplementary Figure S1. For LFD detection, the FIP primer was 5′-biotin labeled. The region between F1c and LB was selected for the designing of a probe for specific detection of the amplicon. The probe was 5′-FITC (Fluorescein Isothiocyanate) labeled.

### 2.5. Optimization of LAMP Conditions

Total DNA extraction from leaves of Chinaberry tree was performed according to the CTAB methods as previously described [13]. The DNA concentration and quality were measured using NanoDrop 2000 (Thermo Scientific, NanoDrop Products, Wilmington, DC, USA). DNA samples were adjusted to a concentration of 500 ng/μL. LAMP reactions were carried out with Bst 2.0 WarmStart DNA polymerase (New England Biolabs, Beijing, China) in a 25 μL volume according to the manufacturer’s instructions. In order to optimize the LAMP reactions, four parameters, i.e., reaction times, temperatures, and the magnesium (Mg^2+^) and dNTP concentrations, were tested and optimized. Reactions were run in a thermocycler (T100 Thermal Cycler, BIO-RAD, Singapore) and terminated with a cycle of 5 min at 4 °C. The LAMP products were analyzed by electrophoresis in 1% agarose gels. For visual observation of the LAMP reactions in microcentrifuge tubes, 0.5 μL of 10,000 ×SYBR Green I (Solarbio, Beijing, China) was added to each microcentrifuge tube containing LAMP products.

### 2.6. LAMP-LFD and Analytical Sensitivity Assay

LAMP-LFD assays were performed with total DNA from healthy leaves or ChTBV1 infected leaves. Lateral flow dipstick (LFD) detections were performed according to the Milenia GenLine HybirDectect REF MGHD1 instruction kit (Milenia Biotec GmbH, Gießen, Germany). Then, 5 μL LAMP products with 2 μL hybridization probes were added into new microcentrifuge tubes containing 100 μL of HybriDetect Assay Buffer (Milenia Biotec GmbH, Gießen, Germany), and the mixture was incubated for 10 min with LFD strips. In order to determine the analytical sensitivity of the ChTBV1 LAMP assays, the plasmid containing the ChTBV1 sequence was adjusted to a concentration of 500 ng/μL and diluted by 10-fold serial dilutions of 1 × 10^0^ to 1 × 10^−10^ and added to conventional PCR or LAMP-LFD reaction systems.

### 2.7. LAMP-LFD Detection in Chinaberry Tree Field Samples

In order to evaluate the practicability of LAMP-LFD detection methods, leaf samples collected from the field were tested by the optimized LAMP-LFD methods. All the amplification products were visualized by SYBR Green I staining, as well as by LFD detection strips.

## 3. Results

### 3.1. Identification of a Novel Badnavirus Infecting Chinaberry Trees

**Figure 1:**
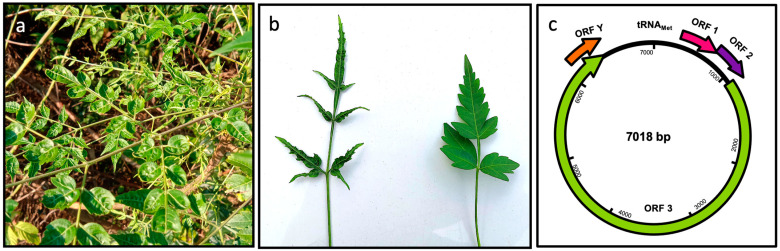
Symptoms of leaf of ChTBV1. (**a**) Leaf deformation observed on a young diseased Chinaberry tree. (**b**) left: Individual leaf showing leaf deformation associated with the presence of ChTBV1. right: typical asymptomatic leaf collected as a negative control. (**c**) Map of the circular dsDNA genome of ChTBV1. Arrows indicate the predicted open reading frames (ORFs).

Deformed leaves of Chinaberry tree (*M. azedarach*) leaves (Figure 1a,b) were collected in Hangzhou, Zhejiang province, during the summer of 2020. In order to identify possible pathogenic viruses associated with the symptoms, an sRNA library from a symptomatic sample was generated and sequenced. Approximately 68.3 million raw reads (averaging 71 nts) were obtained using the Illumina platform. After removing the adapters and low-quality reads, a dataset of 10.2 million unique reads was generated. The resulting sequences were analyzed using VirusDetect, a program for the efficient identity of plant viruses and viroids from deep sRNA sequences [12].

Contig assemblies and BLASTx analyses against NCBI reference virus genomes led to the identification of amino acid sequences with similarity to a polyprotein (GenBank: ASG91886.1) encoded by Cacao swollen shoot virus (CSSV, GenBank accession no. NC_001574) [15]. A total of 15 sequences, with lengths ranging from 60 to 141 nts, had 54–85% amino acid identity with the CSSV polyprotein at a 26.4% query coverage. These results indicated that the Chinaberry trees were infected with a novel badnavirus, which we provisionally named Chinaberry tree badnavirus 1 (ChTBV1).

### 3.2. Complete Genome Sequence of ChTBV1

In order to confirm the presence of ChTBV1, total DNA samples extracted from symptomatic leaves were used as templates for PCR amplification. Then, six specific primer sets (shown in Table S1) covering the entire circular genome were designed based on the assembled contigs (CONTIG 141, CONTIG 205, CONTIG 156, CONTIG 98, CONTIG 200, CONTIG 133), and the PCR amplicons were sequenced using the Sanger method. The DNA fragment assembly resulted in a 7018 nt sequence corresponding to the complete ChTBV1 genome (GenBank accession no. OL630968). BLASTn comparisons of the assembled full-length sequence of the ChTBV1 genome were conducted with badnavirus sequences in the GenBank dataset. These comparisons indicated that ChTBV1 shares 70.8% identity with Cacao swollen shoot Ghana L virus isolates GCR329-14 at a query coverage of 57% (GenBank accession no. NC_040552.1). As with all badnaviruses, the genome of ChTBV1 has a conserved noncoding region containing a putative tRNAmet-binding site (5′-TGG TAT CAG AGC TTC GGC-3′) required for badnavirus replication (Figure 1c).

### 3.3. Genome Organization and Phylogenetic Analyses

The ORF Finder program identified four ChTBV1 encoded ORFs, the position and orientations of which are indicated in Figure 1c and Table 2. ORF1 (nt position 252–683) encodes a putative protein of approximately 16.5 kDa containing a domain of an unknown function (DUF1319) restricted to members of the genus *Badnavirus*. The ORF1-encoded protein shares 67.13% amino acid sequence identity with the ORF1 protein of Cacao swollen shoot Ghana L virus (NCBI protein ID: YP_009551938.1). ORF2 (nt position 680–1108) encodes a 16.1-kDa protein whose sequence overlaps with the 3′ end of the ORF1. Pairwise comparisons suggest that the protein encoded by ORF2 has the highest identity (51.7% at the amino acid level) to its counterpart in Cacao swollen shoot Ghana L virus (NCBI protein ID: YP_009551939). ORF3 (positions 1071–5577). The putative 213 kDa polyprotein contains conserved regions encoding a viral aspartic protease (AP), a zinc knuckle finger, a reverse transcriptase (RT), and RNase H domains and shares 57.63% amino acid sequence identity with the ORF3 of Cacao swollen shoot Ghana L virus (NCBI protein ID: YP_009551940.1). Pairwise alignments using only the RT + RNase H regions of badnavirus sequences currently available at NCBI reveal relatedness to Cacao red vein-banding virus isolate NIG16 (GenBank accession no. MH785303), with the highest identity of 71% at the nt level. ORFY (nt position 6278–6670) is predicted to encode a 14.3-kDa protein whose nt sequence overlaps with the 3′ end of ORF3 by 393 nt. This sequence region had no significant similarity to other badnaviruses at the amino acid level. Based on current ICTV species demarcation limits of 20% differences in the polymerase (RT + RNase H region), ChTBV1 is proposed to be a new member of the *Badnavirus* genus.

**Figure 2:**
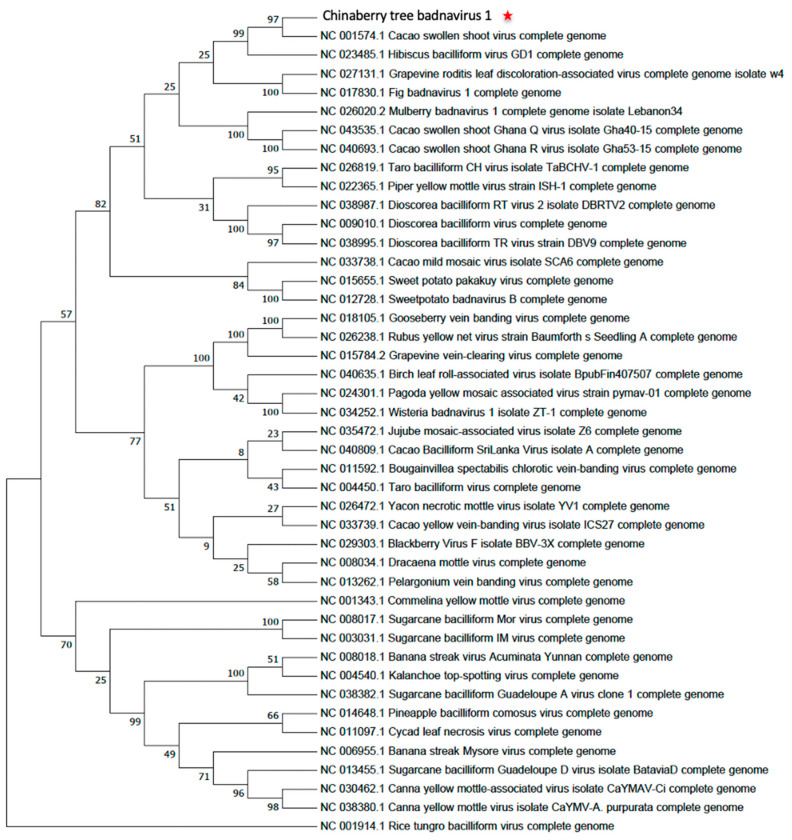
Neighbor-joining phylogenetic trees reconstructed from alignments of the complete genomic nucleotide sequences of ChTBV1 (Red star) with representative members of genus *Badnavirus*, family *Caulimoviridae*. The trees were constructed using the MEGA7 program (Kumar et al., 2016). Bootstrap percentage values determined from 1000 replicates are indicated at the branch internodes. GenBank accession numbers are indicated.

In order to gain further understanding of the genetic relationships of ChTBV1, the MATFF alignment tool (EMBL-EBI) was used for multiple sequence alignments and phylogenetic comparisons of the complete ChTBV1 genomes of the 43 members within the two major badnaviruses clades. Maximum-likelihood phylogenetic analyses were carried out with MEGA 8.0 software. Based on the sequence identity matrix of the complete genome and derived phylogenetic trees, ChTBV1 clusters with Cacao swollen shoot virus in a single sister clade (Figure 2).

### 3.4. Standardization and Optimization of LAMP Reaction Conditions

**Figure 3:**
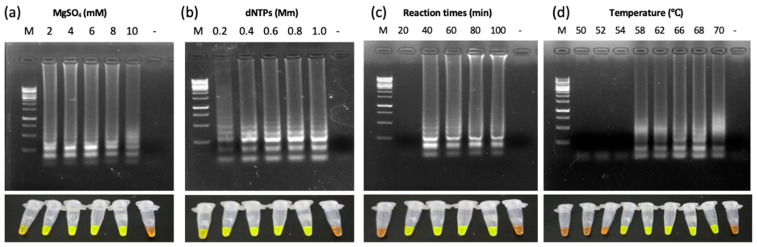
Optimization of LAMP reaction in detection of ChTBV1. (**a**) MgSO4 concentration; (**b**) dNTPs concentration; (**c**) reaction times; (**d**) reaction temperatures; (Lane M: GeneRuler 1 kb Plus DNA Ladder; lane -: negative control (sterile ddH_2_O)).

The reaction reagents used for ChTBV1 LAMP assays were similar to those described in previous studies [6,7,8,9]. Since LAMP amplification successes are closely related to the reaction conditions [16], these were optimized to control variations. The optimum assay conditions were evaluated by both gel electrophoresis and SYBR Green I staining. Firstly, the LAMP assays were conducted at different MgSO4 concentrations (2, 4, 6, 8, and 10 mM). The results showed that green fluorescence and ladder-like bands were evident from 4 mM to 10 mM MgSO4, and the reactions generated distinct ladder-like bands and green fluorescence at 6 mM MgSO4 (Figure 3a). Secondly, different concentrations of dNTPs were used in optimization, and the results revealed typical ladder-like electrophoresis bands, as well as green fluorescence under UV light at dNTP concentrations between 0.2 and 1.0 mM (Figure 3b). The ladder-like electrophoresis bands were clearer at 0.8 mM dNTPs than at other concentrations. The appropriate temperatures and times correlated with activation of enzymes and reaction stabilities. The temperature-optimization assays showed that LAMP amplification products could be generated from 58 ◦C to 70 °C. In addition, the time-optimized results indicated that the amplification products from 40 min to 100 min were the most clearly detected after agarose gel electrophoresis and adding SYBR Green I dye. Therefore, a 68 °C temperature and 40 min reaction time were selected for the assays (Figure 3c,d). In summary, the optimal system consisted of a total reaction volume of 25 μL containing 2.5 μL of 10 ×Isothermal Amplification Buffer, 0.8 mM dNTPs, 6 mM MgSO_4_, 0.2 μM outer primers (F3, B3), 1.4 μM inner primers (FIP, BIP), 0.4 μM LB primer, 8U Bst 2.0 WarmStart DNA polymerase (New England Biolabs, Beverly, MA, USA), 1 μL target DNA, and ddH_2_O added to 25 μL. The mixture was then incubated at 68 °C for 40 min.

### 3.5. Analytical Specificity and Sensitivity of the LAMP-LFD Method

**Figure 4:**
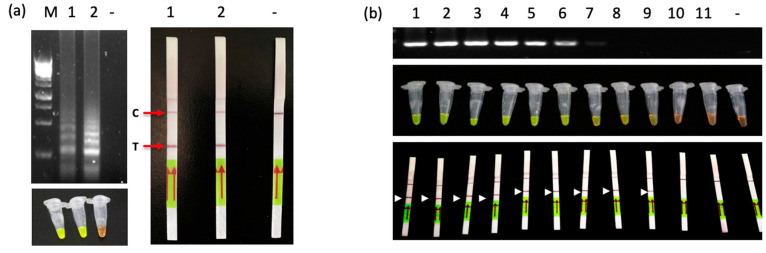
Analytical specificity and sensitivity of LAMP-LFD assays in detecting ChTBV1. (**a**) Establishment of a ChTBV1 LAMP-LFD assay. Top left panel: LAMP products visualized by gel electrophoresis; Bottom left panel: LAMP products visualized with SYBR Green I dye; Right panel: LAMP products visualized with LFD strips. Lane M: GeneRuler 1 kb Plus DNA Ladder; Lane1: DNA extracted from ChTBV1 infected leaves. Lane 2: Plasmid containing the ChTBV1 sequence A1. Lane -: DNA extracted from uninfected leaves. C: control band; T: test band. (**b**) LAMP-LFD detection limits for ChTBV1 dilutions. Top panel: PCR assay sensitivity test; Middle panel: SYBR Green I-visual LAMP sensitivity test; Bottom panel: LAMP-LFD sensitivity test. M: GeneRuler: 1 kb DNA Ladder; Lanes 1 to 11: Ten-fold serial dilutions (10 ^0^ to 10^−10^) of plasmid containing the ChTBV1 sequence (0.5 μg/μL); lane -: Negative template control (sterile ddH_2_O).

The analytical specificity of the developed LAMP-LFD assay was evaluated using ChTBV1 infected leaves, a plasmid containing ChTBV1 sequence, and healthy tissue as templates. Agarose gel electrophoresis results indicated that the LAMP products appearing as ladder-like bands were present in both ChTBV1 infected leaf extracts and plasmid reactions containing ChTBV1 sequence, but not in healthy tissue extracts. The visual detection results by adding SYBR Green I dye under UV light were also consistent with the gel electrophoresis results. In addition, the FITC-labeled green fluorescence LAMP product was only detected in samples containing ChTBV1 template DNA (Figure 4a). Further, the LFD test results showed that the set of LAMP primers were specific to ChTBV1 detection (Figure 4a). Two obvious bands were visualized both in the test and negative control lines, but only the control bands were evident in the negative control samples (Figure 4a).

The analytical sensitivity of LAMP-LFD detection was evaluated using a 10-fold serial dilution of DNA. The plasmid containing the ChTBV1 sequence was adjusted to a concentration of 500 ng/μL. The results showed that the detection limit of the LAMP method was 10^−8^ dilutions (5 fg) by both SYBR Green I staining and LFD visual detection. In contrast, the conventional PCR method could detect the virus at the 10^−5^ dilutions (5 pg) and produced a very weak band at the 10^−6^ dilutions (0.5 pg) (Figure 4b). These results indicate that the detection sensitivity of the LAMP-LFD was about 10^2^-fold higher than the conventional PCR method.

### 3.6. Application of the LAMP-LFD Method for Detection in Field Samples

**Figure 5:**
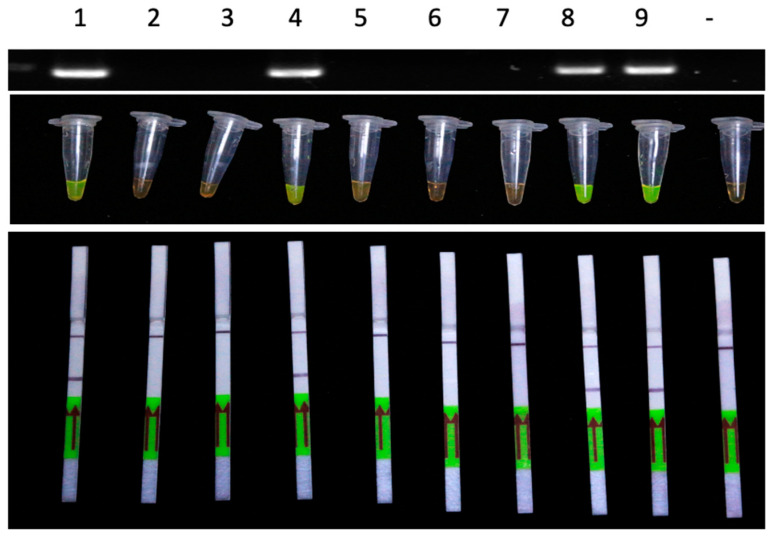
LAMP-LFD assay detection of ChTBV1 in Chinaberry tree field samples collected from Zhejiang Province. Top Panel: RT-PCR products separated by agarose gel electrophoresis. Middle Panel: LAMP products visualized with SYBR Green I dye; Bottom Panel: LAMP products visualized in LFD strips. Lanes 1–9: Chinaberry tree leaves from field samples; Lane -: negative template control (sterile ddH_2_O).

In order to further investigate the applicability of the LAMP-LFD method for detection of ChTBV1 in Chinaberry trees, nine Chinaberry leaf samples from different trees in a botanical garden in Hangzhou, Zhejiang province were tested by the LAMP-LFD method. Four samples (Figure 5 samples 1, 4, 8, and 9) with leaf deformation symptoms were positive, whereas the other five asymptomatic samples were ChTBV1-free, indicating that ChTBV1 infections are prevalent in Hangzhou. This result was also confirmed by conventional PCR. *In total*, these results indicate that the LAMP-LFD method can be used successfully to detect ChTBV1 in field samples.

At the same time, we noticed that the online trading of tree seedlings in China was becoming more and more common. We searched China’s popular online trading websites, including Taobao and JD.com, using “ 苦楝 ” (Chinaberry tree in Chinese) as the keyword, and found that dozens of companies are engaged in online trading of Chinaberry tree seedlings. These companies are concentrated in Guangxi, Guangdong, Jiangsu, and Henan, among other provinces. In order to investigate the presence of the virus in several provinces of China, we purchased 60 Chinaberry trees (one or two years old) from Guangxi, Guangdong, Jiangsu, Anhui, Shandong, and Henan provinces through Taobao and JD.com. Using the virus detection methods established in this study, we detected positive ChTBV1 results in two Chinaberry tree seedlings from Jiangsu province. This result indicates that ChTBV1 distribution is not limited to Zhejiang province but is also found in the neighboring Jiangsu province.

## 4. Discussion

The study described here has identified and characterized a new badnavirus from deformed leaves of Chinaberry tree and provisionally named ChTBV1. The full-length genome of the ChTBV1 was obtained by PCR and sequenced by Sanger sequencing, indicating that the virus is likely to exist in the host as an episomal virus. ChTBV1 shares 70.8% identity with Cacao swollen shoot Ghana L virus (CSSV) isolate GCR329-14 at a query coverage of 57%. The genome organization of ChTBV1 is similar to that of CSSV (Figure 1), and the virus has a close phylogenetic relationship with CSSV, based on analyses of nucleotide sequences of their complete genomes (Figure 2). According to the species classification criteria of the 10th report of the International Committee on Taxonomy of Viruses [17], ChTBV1 should be considered a new member of the genus *Badnavirus* in the family *Caulimoviridae*.

A majority of badnaviruses infect hosts that are propagated vegetatively, and few are known to be seed-transmitted [3]. However, attempts to transmit the virus to healthy Chinaberry trees mechanically or by grafting were unsuccessful (data not shown). Therefore, it remains unclear if the leaf deformation symptom is caused by ChTBV1. The secondary or horizontal spread of the badnavirus species occurs through various mealybug species or aphids [3]. It was reported that fifty-six species of arthropods were found on members of the genus *Melia* [18]. Among them, two monophagous leafhoppers, *Elbelus melianus* Kuoh [19] and *Erythroneura melia* Kuoh [20], cause significant damage to Chinaberry trees. It needs to be further investigated whether the virus is spreading through these insect vectors.

Through analysis of NGS results, we only identified the siRNA origin of ChTBV1, and no other viral siRNAs were detected in the symptomatic sample. Furthermore, virus detection analyses showed that all symptomatic samples tested positive for ChTBV1 infection, whereas asymptomatic samples were ChTBV1-free, suggesting a possible association between the virus and the symptoms observed. The ability to determine the infection risks of ChTBV1 earlier in the transplantation of Chinaberry tree seedlings would enable timely and effective prevention of virus spreading. In field applications, diagnostic technologies with the highest sensitivity and specificity should be provided. At the same time, low-cost, fast, and easy applications should be considered. Classic detection methods for plant viruses, such as polymerase chain reaction (PCR), real-time PCR, and enzyme-labeled immuno-sorbent assay (ELISA), are helpful for plant virus detection but are costly, time-consuming, and require skilled technicians [9]. Comparatively, LAMP only requires a constant temperature device that is easy to operate, convenient, and efficient. For this purpose, a rapid LAMP-LFD visualization method was established that requires only minimal equipment and technical training for application. The technique permitted sample DNA to be amplified at 68 °C for 40 min and hybridized with a ChTBV1 probe for 5 min and is about 100-fold more sensitive than conventional PCR. We successfully applied the LAMP-LFD method for the detection of ChTBV1 in nine leaf samples collected in Zhejiang and 60 samples from six other provinces that have online trading sites for Chinaberry tree seedlings. The positive results of samples from Jiangsu province indicate that ChTBV1 is not confined to Zhejiang and that online trading could be a potential source for the widespread distribution of the virus.

In conclusion, we identified and characterized a new viral pathogen designated Chinaberry tree badnavirus 1 (ChTBV1) from Chinaberry trees in China and developed a visual LAMP-LFD method for the rapid detection. This method was successfully applied for the detection of ChTBV1 in leaf samples from fields and online distribution sites. We believe that this approach can be deployed for effective field-testing to facilitate timely and effective ChTBV1 diagnostics and restriction of disease spread.

## Figures and Tables

**Table 1:** List of primers used for detection of ChTBV1 by LAMP.

Primer Type	Primer Name	Sequence (5′–3′)	Length (bp)	Usage
PCR	ChTBV1-F	CTGCAGCTTCTGCAAGAG	18	PCR Detection of ChTBV1
	ChTBV1-R	GAAAATGTAGGGCTCATTGT	20	
LAMP	F3	GAGAAATGGAGAAACAAACGT	21	LAMP Detection of ChTBV1
	B3	CAACCTTTCCAAATCTCTGT	20	
	LB	TCGCTAGAGGATCCTGCTAC	21	
	FIP (F1c + F2) ^a^	GTCCCTTCTCTTCCCTCAGCGAGAATGATGTATCCCACGG	40	
	BIP (B1c + B2)	CTGTCCCAGATAAGAAGAGTGTTCCCTGATCCTGAATATGTGTTGT	46	
	Probe ^b^	GGACAGAATATTCTGTGTC	19	

Note ^a^: A 5′-biotin-labeled FIP primer was used in the RT-LAMP-LFD assay for PCR detection of ChTBV1. Note ^b^: A 5′-fluorescein isothiocyanate (FITC)-labeled BIP primer was used in the RT-LAMP-LFD assay for detection of ChTBV1.

**Table 2:** Putative open reading frames (ORF) in the ChTBV1 genome sequence.

ORF	Genome Position (5′-3′)	Protein		Function	Top Two Viruses with the Highest Amino Acid Sequence Identity
		Amino Acid	kDa		
1	252–683	144	16.5	Unknown	67.1%, ORF1 of Cacao swollen shoot Ghana L virus at a query coverage of 99%; 61.5%, hypothetical protein of Cacao swollen shoot virus at a query coverage of 99%.
2	680–1108	143	16.1	Unknown	51.7%, ORF2 of Cacao swollen shoot Ghana L virus at a query coverage of 99%; 42.9%, ORF2 of Cacao swollen shoot Ghana K virus at a query coverage of 98%.
3	1071–6647	1858	213	Polyprotein	57.6%, ORF1 of Cacao swollen shoot Ghana L virus at a query coverage of 99%; 56.1%, ORF1 of Cacao swollen shoot virus at a query coverage of 97%.
Y	6278–6670	130	14.3	Unknown	-

## Supplementary Materials

The following are available online at https://www.mdpi.com/article/10.3390/v13122408/s1, Figure S1: The locations of LAMP primers in the ChTBV1 ORF3 sequence, Table S1: List of primers used for cloning of the ChTBV1 genome.
