# Supplementary material for: Identification of a New Badnavirus in the Chinaberry (Melia azedarach) Tree and Establishment of a LAMP-LFD Assay for Its Rapid and Visual Detection
Source: Viruses. 2021 Dec 1;13(12):2408. doi: 10.3390/v13122408 (PMC8704090; doi:10.3390/v13122408)
Supplement: Supplementary file 1 [file viruses-13-02408-s001.zip › viruses-1473293-supplementary.pdf]

Table S1. List of primers used for cloning of the ChTBV1 genome.

Primer	Sequences (5′–3′)
ChTBV1/F1/F	GGGTAAGTCTTGTTGATTACATTACTG
ChTBV1/F1/R	GAGGCTTTCTGGCTGTATGAAT
ChTBV1/F2/F	TCTAGTAACTGACGGAAACCAGC
ChTBV1/F2/R	CCAAATCTCTGTATGCCTGATC
ChTBV1/F3/F	GTGTTCTCGCTAGAGGATCCT
ChTBV1/F3/R	CCGATAGACTGTATATGGCATCAG
ChTBV1/F4/F	AGATTGTATCTGTGCAGGACG
ChTBV1/F4/R	CTGTATTTTCCCTCCAAGATCCATAG
ChTBV1/F5/F	AGTAGGAGGGCATAATTTCAGAATC
ChTBV1/F5/R	CCATCTGGAACCCAAAATGCT
ChTBV1/F6/F	AGCCCAGATATTTTCCAAGTTTGAT
ChTBV1/F6/F	CTGCTAACTGGTTCTCCTTTCC

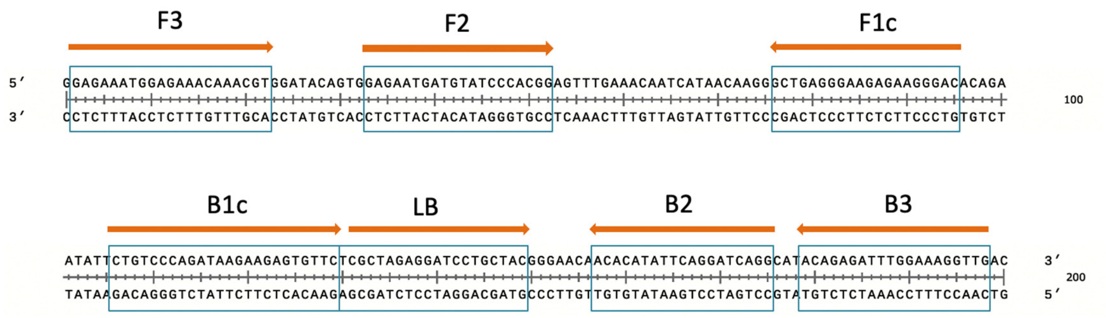

Figure S1. The locations of LAMP primers in the ChTBV1 ORF3 sequence. The arrows showed the positions and directions of two outer primers (F3, B3), a forward inner FIP primer (F1c + F2), a reverse inner BIP primer (B1c + B2), and loop primer (LB).
